# Platelet-lymphocyte ratio acts as an indicator of poor prognosis in patients with breast cancer

**DOI:** 10.18632/oncotarget.13714

**Published:** 2016-11-30

**Authors:** Yanyun Zhu, Wen Si, Qiong Sun, Boyu Qin, Weihong Zhao, Junlan Yang

**Affiliations:** ^1^ Department of Medical Oncology, Chinese PLA General Hospital, Beijing 100853, China; ^2^ Department of Medical Oncology, Beijing Shijitan Hospital, Capital Medical University, Beijing 100038, China

**Keywords:** breast cancer, prognosis, biomarker, meta-analysis, PLR

## Abstract

Platelet-lymphocyte ratio (PLR) is a hematological parameter which is investigated as a biomarker for prognosis in patients with breast cancer. Due to the controversial results from previous studies, we performed a meta-analysis. Databases of PubMed, Embase and Web of Science were searched to identify eligible studies. STATA version 12.0 was used for statistical analysis. Seven studies with 3,741 patients were ultimately included in this meta-analysis. High PLR was associated with poor overall survival (OS) (HR = 1.55, 95% CI = 1.07–2.25, *p* = 0.022) and disease-free survival (DFS) (HR = 1.73, 95% CI = 1.3-2.3, *p* < 0.001) in breast cancer patients. Subgroup analyses disclosed that elevated PLR could predict worse OS in Asian populations and poor DFS in both Asian and non-Asian patients. In addition, PLR remains a significant prognostic marker for OS in patients receiving systemic treatment (HR = 1.78, 95% CI = 1.06–2.99, *p* = 0.03) and patients receiving chemotherapy (HR = 2.82, 95% CI = 1.09–7.26, *p* = 0.032). High PLR also indicates poor DFS in patients who receive chemotherapy (HR = 2.6, 95% CI = 1.47–4.61, *p* = 0.001), surgery (HR = 1.8, 95% CI = 1.12–2.89, *p* = 0.016) and systemic treatment (HR = 2.03, 95% CI = 1.03–4.01, *p* = 0.042). Moreover, PLR was also in association with HER-2 positivity (OR = 1.48, 95% CI = 1.2–1.83, *p* < 0.001). In conclusion, this meta-analysis revealed that PLR could serve as an indicator of poor prognosis in patients with breast cancer.

## INTRODUCTION

Breast cancer is the most commonly diagnosed cancer and the leading cause of cancer mortality in women worldwide [1]. In the past decade, the incidence and mortality of breast cancer is still gradually increasing [2]. Therapeutic approaches including chemotherapy, radiotherapy, and hormonal therapy are applied in clinical practice for breast treatment. However, the long term survival outcomes are still suboptimal, especially for high-risk individuals [3]. Prognostic factors play important roles in risk estimation and treatment responses prediction for cancer patients. For patients with breast cancer, tumor size, lymph node status, histological grade, and hormone receptor status are commonly used prognostic markers. However, the discriminant efficiency of most prognostic biological factors is still lack of accuracy which reflects the fact that easily available and efficient prognostic variables are required.

Recently, inflammatory responses in tumor microenvironment have been shown to be associated with tumor progression and metastases [4]. Cancer-related inflammatory responses and assist cancer cells in the processes of proliferation, infiltration, neovascularization, and dissemination [5]. Some hematological biomarkers are easy available and costless because they are derived from laboratory tests. There parameters include C-reactive protein (CRP), Glasgow Prognostic Score (GPS), platelet- lymphocyte ratio (PLR), and neutrophil-lymphocyte ratio (NLR). PLR is calculated as platelet counts divided by lymphocyte counts. PLR is reported to be correlated with worse outcomes in different malignant tumors such as colorectal cancer [6], lung cancer [7, 8], and gastric cancer [9, 10]. Growing evidence also showed that PLR could provide implications for therapeutic modalities selection and prognosis prediction for breast cancer patients [11–13]. However, the association between PLR and breast cancer prognosis is controversial because relevant studies present different results [14–16]. These discrepancies could be caused by different study design and small sample sizes. Therefore, in this study, a meta-analysis was performed to reveal the impact of PLR on survival and clinical characteristics in breast cancer.

## RESULTS

### Search results and study characteristics

**Figure 1:**
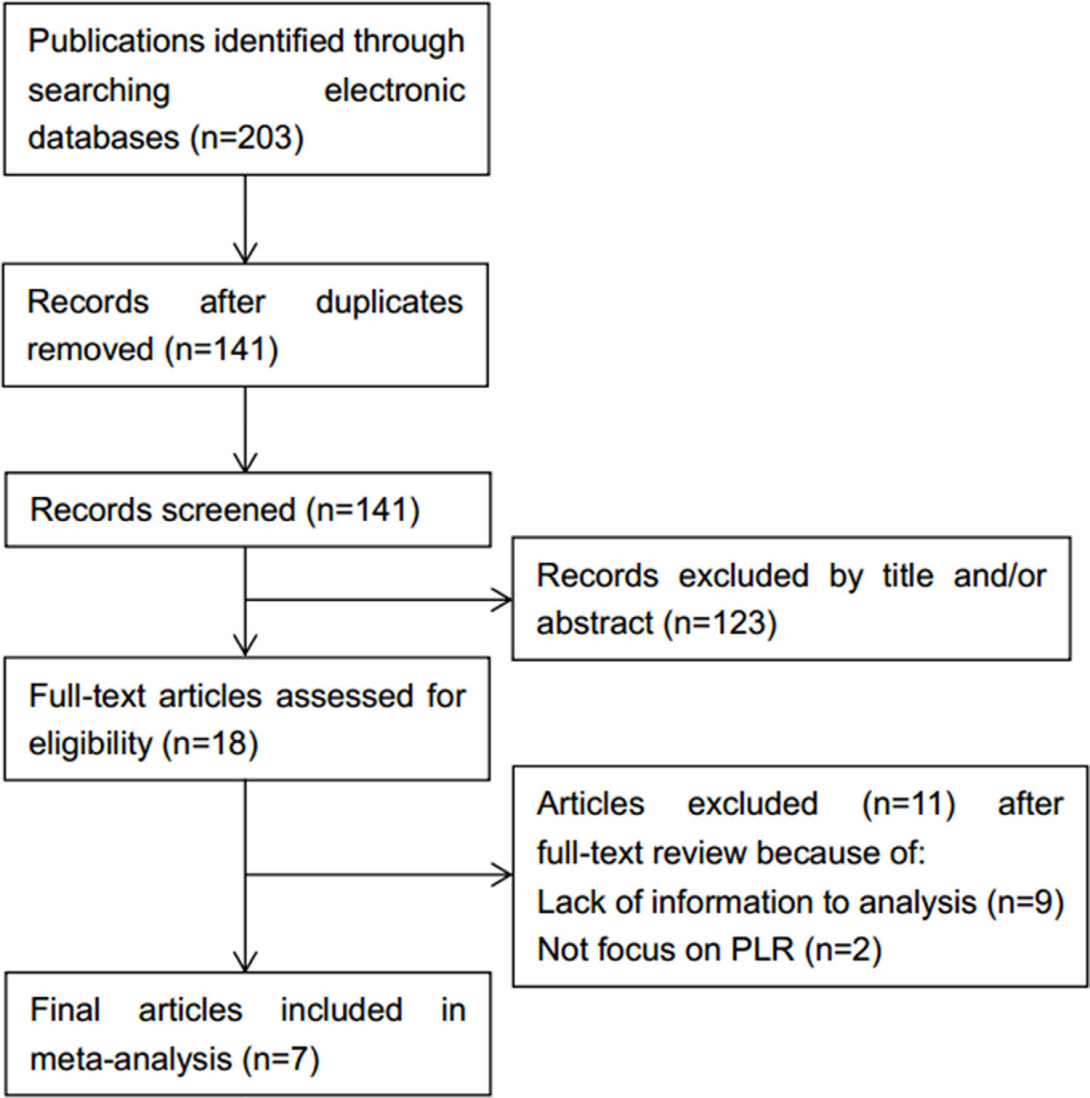
Methodological flow diagram of the meta-analysis

Initially, 203 records were identified from electronic databases. After removal of duplicates and inspection of titles and/or abstracts, 18 full-text articles were further evaluated. Of these 18 studies, 11 were excluded because they were studies with inadequate data or did not report data on PLR. As a result, 7 studies [14–20] involving 3,741 patients were enrolled in this study. Detailed search steps were described in Figure 1. The sample sizes vary from 62 to 1,435 per study with a median value of 437. Five studies [14, 16, 17, 19, 20] were conducted in Asian countries and two studies [15, 18] were carried out in non-Asian countries. The cut-off values for PLR ranged from 110 to 292. Six studies [14–19] reported the correlation between PLR and OS and five studies [15–17, 19, 20] investigated the association between PLR and DFS. The NOS scores of all studies were more than 7. General features of the 7 included studies were summarized in Table 1.

**Table 1:** Main characteristics of included studies in meta-analysis

Study	Year	Region	Sample size	Research period	Stage	Treatment	Cut-off value	Outcome	NOS score
Asano	2016	Japan	177	2007–2013	II–IV	Chemotherapy	150	OS, DFS	8
Azab	2013	USA	437	2004–2006	I–IV	Systemic treatment	185	OS	7
Cihan	2014	Turkey	350	2005–2010	I–III	Radiotherapy	160	OS, DFS	8
Gunduz	2015	Turkey	62	2008–2010	I–III	Chemotherapy	200	DFS	8
Hong	2016	China	487	2009–2010	I–III	Surgery	110	OS, DFS	8
Koh	2015	Malaysia	1435	2000–2008	I–IV	Systemic treatment	185	OS	7
Krenn-Pilko	2014	Austria	793	1999–2004	I–III	Systemic treatment	292	OS, DFS	8

### Impact of PLR on OS and DFS in breast cancer

**Figure 2:**
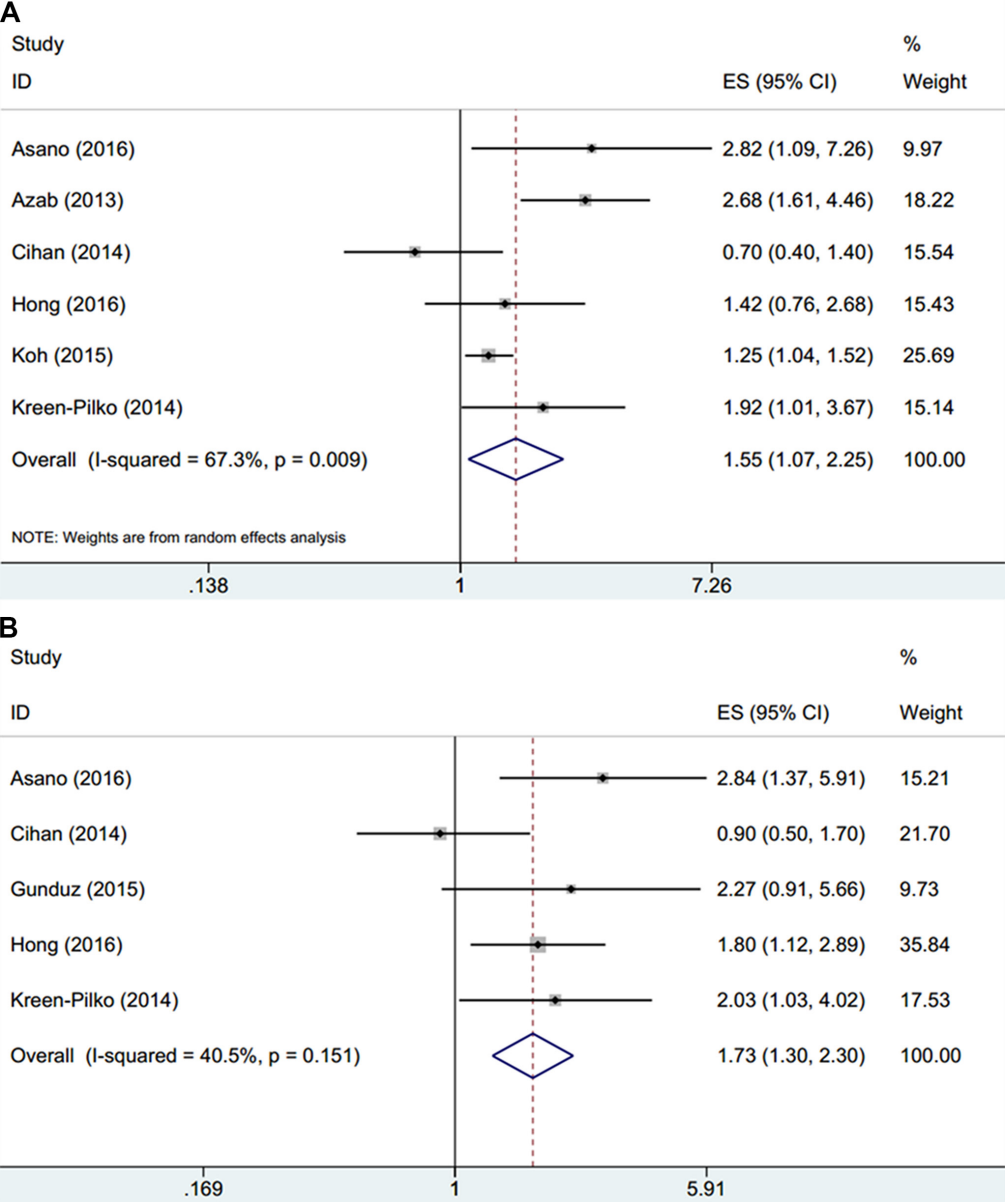
Forrest plots of studies evaluating HRs of the PLR for (**A**) OS and (**B**) DFS

HRs and 95% CIs from 6 studies [14–19] comprising 3,679 patients were extracted and pooled. Random-effects model was used due to significant heterogeneity (*I*^2^ = 67.3%, P_h_=0.009, Table 2, Figure 2A). The pooled results were HR = 1.55, 95% CI = 1.07 2.25, *p* = 0.022. We also conducted subgroup analysis for further investigation. The results showed that PLR was still an indicator for poor OS in non-Asian patients (HR = 2.36, 95% CI = 1.58–3.52, *p <* 0.001) and in studies with sample sizes > 400 (HR = 1.67, 95% CI = 1.13–2.47, *p* = 0.01). In addition, PLR remains a significant prognostic marker for OS in patients receiving systemic treatment (HR = 1.78, 95% CI = 1.06–2.99, *p* = 0.03) and patients receiving chemotherapy (HR = 2.82, 95% CI = 1.09–7.26, *p* = 0.032). A total of 5 studies [15–17, 19, 20] containing 1,869 patients reported the prognostic significance of PLR on DFS. The pooled results showed that PLR was significantly associated with worse DFS (HR = 1.73, 95% CI = 1.3–2.3, *p <* 0.001) and the heterogeneity was not significant (*I*^2^ = 40.5%, *P*_h_ = 0.151, Table 2, Figure 2B). Subgroup analysis demonstrated that PLR was connected with shorter DFS in both Asian patients (HR = 1.72, 95% CI = 1.06–2.8, *p* = 0.027) and non-Asian countries (HR = 2.03, 95% CI = 1.03–4.01, *p* = 0.042) and in studies with patients amount > 400 (HR = 1.87, 95% CI = 1.27–2.76, *p* = 0.002). Moreover, high PLR also indicates poor DFS in patients who receive chemotherapy (HR = 2.6, 95% CI = 1.47–4.61, *p* = 0.001), surgery (HR = 1.8, 95% CI = 1.12–2.89, *p* = 0.016) and systemic treatment (HR = 2.03, 95% CI = 1.03–4.01, *p* = 0.042). These results indicated that high PLR was significantly associated with poor OS and DFS in patients with breast cancer.

**Table 2:** Meta-analysis of PLR and OS, DFS

Factors	No. of studies	No. of patients	HR (95% CI)	*p*	Effects model	Heterogeneity	
						*I*^2^ (%)	*P*_h_
Overall for OS	6	3,679	1.55 (1.07–2.25)	0.022	Random	67.3	0.009
Region							
Asian	4	2,449	1.26 (0.85–1.85)	0.251	Random	52.2	0.099
Non-Asian	2	1,230	2.36 (1.58–3.52)	< 0.001	Fixed	0	0.427
Treatment							
Systemic treatment	3	2,665	1.78 (1.06–2.99)	0.03	Random	76.6	0.014
Chemotherapy	1	177	2.82 (1.09–7.26)	0.032	–	–	–
Radiotherapy	1	350	0.7 (0.37–1.31)	0.264	–	–	–
Surgery	1	487	1.42 (0.76–2.68)	0.273	–	–	–
Sample size (*n*)							
> 400	4	3,152	1.67 (1.13–2.47)	0.01	Random	64.8	0.036
< 400	2	527	1.34 (0.34–5.23)	0.674	Random	82.7	0.016
Overall for DFS	5	1,869	1.73 (1.3–2.3)	< 0.001	Fixed	40.5	0.151
Region							
Asian	4	1,076	1.72 (1.06–2.8)	0.027	Random	53.6	0.091
Non-Asian	1	793	2.03 (1.03–4.01)	0.042	–	–	–
Treatment							
Chemotherapy	2	239	2.6 (1.47–4.61)	0.001	Fixed	0	0.708
Radiotherapy	1	350	0.9 (0.49–1.66)	0.736	–	–	–
Surgery	1	487	1.8 (1.12–2.89)	0.016	–	–	–
Systemic treatment	1	793	2.03 (1.03–4.01)	0.042	–	–	–
Sample size (*n*)							
> 400	2	1,280	1.87 (1.27–2.76)	0.002	Fixed	0	0.773
< 400	3	589	1.73 (0.81–3.72)	0.158	Random	68.4	0.042

OS = overall survival; DFS = disease-free survival.

### Relationships between PLR and clinicopathological features

**Figure 3:**
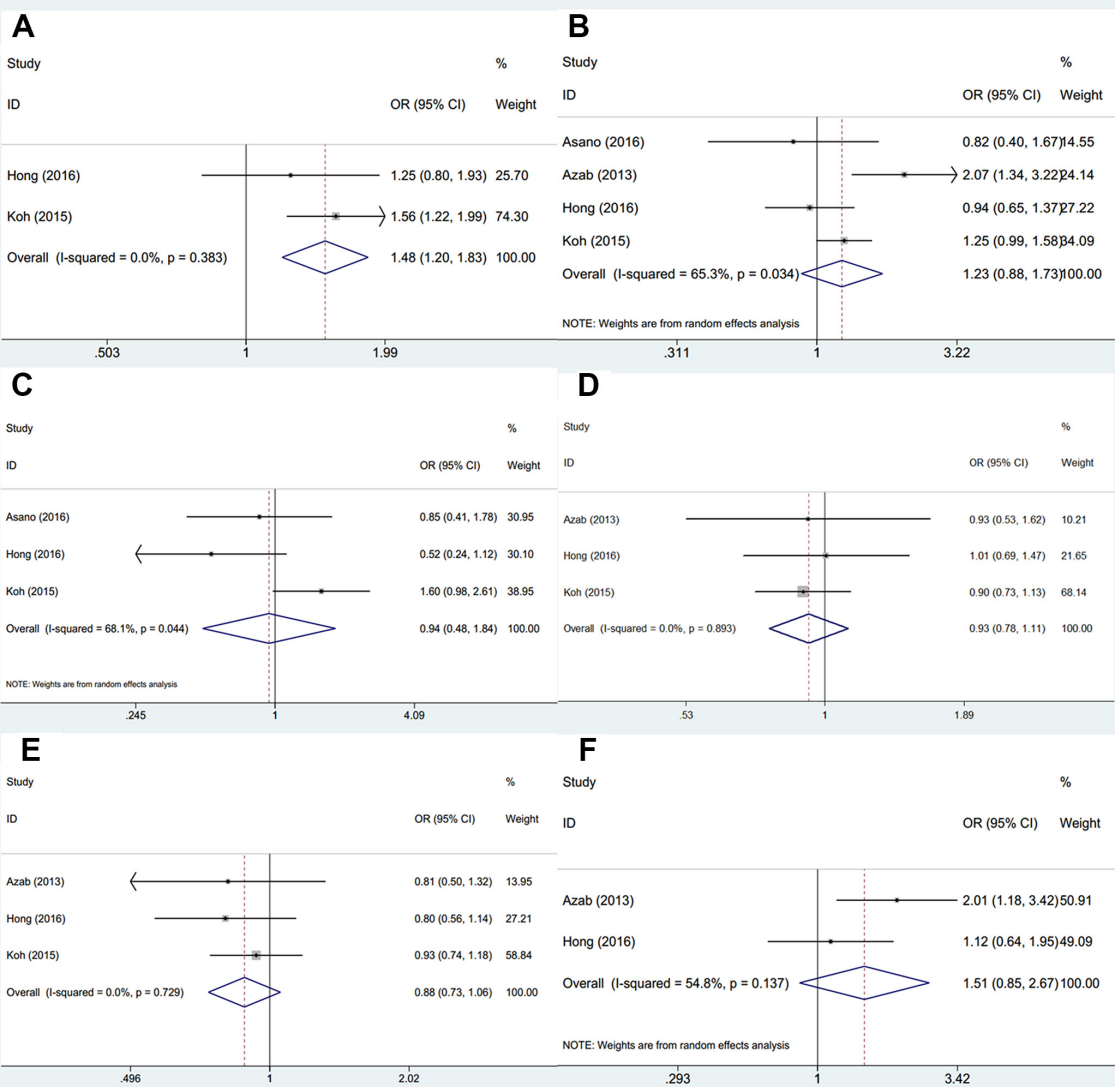
Forrest plots of associations between PLR and (**A**) HER-2 status; (**B**) Lymph node metastasis; (**C**) Unclear grade; (**D**) ER status; (**E**) PR status and (**F**) AJCC stage

We explored the correlation between PLR and 6 clinicopathological parameters. As shown in Figure 3, PLR was shown to be associated with human epidermal growth factor receptor-2 (HER-2) positivity (OR = 1.48, 95% CI = 1.2–1.83, *p* < 0.001). However, the pooled data demonstrated that PLR was not significantly correlated with other 5 clinicopathological factors including lymph node metastasis (OR=1.23, 95% CI = 0.88–1.73, *p* = 0.229), unclear grade (OR=0.94, 95% CI = 0.48–1.84, *p* = 0.859), estrogen receptor (ER) status (OR=0.93, 95% CI = 0.78–1.11, *p* = 0.42), progesterone receptor (PR) status (OR= 0.88, 95% CI = 0.73–1.06, *p* = 0.168) or AJCC stage (OR=1.51, 95% CI = 0.85–2.67, *p* = 0.158).

### Publication bias

We performed Begg's funnel plot and Egger's linear regression test to estimate potential publication bias in this meta-analysis. The *p* values for OS were 0.452 (Begg's test) and 0.418 (Egger's test) and *p* values for DFS were 0.221 (Begg's test) and 0.583 (Egger's test). The results showed that there was no significant publication bias in our study.

## DISCUSSION

In this meta-analysis containing 7 studies, the combined results showed that PLR was a significant biomarker for poor OS (HR = 1.55, 95% CI = 1.07–2.25, *p* = 0.022) and DFS (HR = 1.73, 95% CI = 1.3–2.3, *p* < 0.001). Subgroup analyses disclosed that elevated PLR could predict worse OS in Asian populations and poor DFS in both Asian and non-Asian patients. In addition, PLR was also in association with HER-2 positivity (OR = 1.48, 95% CI = 1.2–1.83, *p <* 0.001). Taken all these into consideration, PLR could serve as a convenient and reliable marker for breast cancer prognostication.

Tumor-promoting inflammation is an emerging hallmark of cancer [21]. Systemic inflammatory responses can facilitate tumor progression in almost every single step including initiation, progression, and metastasis [5]. On the one hand, current evidence shows that platelets can guard tumor cells from immune elimination and are involved in development of aggressive tumor behaviors [22]. In addition, platelets can promote tumor-cell transendothelial migration and metastasis through the mediation of P2Y_2_ receptor [23]. Platelets can secret a variety of growth factors including platelet derived growth factor (PDGF) [24], platelet-activating factor (PAF) [25], and vascular endothelium growth factor (VEGF) [26], which could further support tumor growth, angiogenesis and metastasis [27]. Therefore, increased platelet counts have negative effects on patient survival. On the other hand, lymphocytes play an important role in tumor-derived inflammatory responses [28]. Lymphocytes have an antitumor activity by inducing cytotoxic cell death and inhibiting tumor proliferation [4]. Several studies reported that the increased infiltration of lymphocytes in tumor tissue predicted better survival outcomes in cancer patients [29, 30].

Notably, previous studies showed that high PLR was in association with poor survival in other tumors including non-small cell lung cancer [31], colorectal cancer [32], gastric cancer [33], and various solid tumors [34, 35] by performing meta-analysis. Our results regarding breast cancer were in line with results of studies on other cancer forms [31, 33, 36]. We noted that breast cancer was investigated merely as a small proportion of all cancer types in previous meta-analyses with at most two primary studies included [34, 35]. There was no study focusing on the prognostic value of PLR on breast cancer through meta-analysis. To our knowledge, the present study is the first meta-analysis to investigate the relationship between PLR and breast cancer.

There are several limitations to our study. First, this meta-analysis was performed based on the pooled HRs and 95% CIs from eligible studies other than detailed individual information. Thus, potential bias may still exist. Second, the cut-off values for PLR were different in included studies, because they identified the cut-off values according to various criteria. Although the patient groups were divided into PLR-high and PLR-low populations, the stratifications may change when the cut-off changes. Therefore, a standard and uniform cut-off value defining high PLR is needed.

In conclusion, this meta-analysis demonstrated that high PLR was an indicator of worse OS and DFS in breast cancer. Moreover, PLR was related to HER-2 positivity. Further high-quality and large-scale studies are required to determine the validation of other results.

## MATERIALS AND METHODS

### Search strategy

This study was conducted referring to the Preferred Reporting Items for Systematic Reviews and Meta-Analyses (PRISMA) statement [37]. Relevant studies were thoroughly searched from PubMed, Embase, Web of Science, and China National Knowledge Infrastructure (CNKI) up to August, 2016. The search strategy included following keywords: “PLR”, “platelet lymphocyte ratio”, “breast cancer”, “breast carcinoma”, and “breast neoplasms” [MeSH Terms]. References in all relevant articles were also checked to identify potentially relevant studies. There was no language restriction.

### Selection criteria

Inclusion criteria were as follows: (1) PLR was measured pretreatment on basis of blood tests; (2) the diagnosis of breast cancer established by pathological examination; (3) reported a cut-off value for PLR; (4) reported the associations between PLR and survival outcomes; (5) sufficient data were provided to calculate hazard ratios (HRs) and 95% confidence intervals (CIs). Exclusion criteria in this study were as follows: (1) reviews, case reports, conference abstracts and letters; (2) studies with insufficient data; (3) animal studies. Two independent investigators (YYZ and WS) evaluated the candidate studies and disagreements were resolved by discussing with a third investigator (JLY).

### Data extraction and quality assessment

Two investigators (YYZ and WS) independently performed the data extraction from eligible studies. The following information was extracted: first author's name, year of publication, country, sample size, research period, survival outcomes, cut-off value, and clinicalpathological characteristics. The qualities of included studies were evaluated using Newcastle–Ottawa Quality Scale (NOS) assessment [38]. Three parts including selection, comparability, and outcomes were evaluated in this scale with a maximum score of 9. Studies with scores ≥ 7 were considered as high-quality studies.

### Statistical analysis

HR and 95% CI were used as the effective measures to estimate the relationships of PLR and overall survival (OS) and disease-free survival (DFS). If possible, HRs and 95% CIs were directly extracted from included studies, or they were computed based on methods by Tierney et al [39]. Odds ratio (OR) and 95% CI were utilized to evaluate the associations between PLR and clinicopathological factors. Heterogeneity among studies was assessed by Cochran's *Q* test and the Higgins' *I*^2^ statistic. A *P* value for heterogeneity < 0.05 and/or *I*^2^ > 50% indicated significant heterogeneity, and a random-effects model was used; otherwise, a fixed-effects model was applied. Publication bias was tested by Begg's funnel plots and Egger's linear regression test. *P* < 0.05 was considered as statistically significant. All analyses were performed by using STATA version 12.0 (Stata Corporation, College Station, TX, USA).
